# Superficial Temporal Artery Pseudoaneurysm: A Case Report

**DOI:** 10.3389/fsurg.2015.00051

**Published:** 2015-10-06

**Authors:** Syed Muneeb Younus, Muhammad Imran, Rabia Qazi

**Affiliations:** ^1^Neurosurgery, Dow University of Health Sciences, Karachi, Pakistan

**Keywords:** pseudoaneurysm, craniotomy, superficial temporal artery, meningioma

## Abstract

Pseudoaneurysms of the superficial temporal artery are an uncommon vascular lesion of the external carotid system and most often the result of blunt head trauma. The frequency of pseudoaneurysms of the superficial temporal artery developing after craniotomy is exceedingly low and only a few cases have been reported. We present a case of pseudoaneurysm of this type in a 45-year-old male who underwent craniotomy for excision of meningioma. One month postoperatively, the craniotomy flap exhibited an enormous diffuse pulsate swelling. The suspected diagnosis of pseudoaneurysm arising from superficial temporal artery was confirmed on angiography. Surgical excision was done and no recurrences of the tumor or aneurysm were noted on subsequent follow up.

## Introduction

A pseudoaneurysm is a dilation of an artery that includes a defect in one or more layers of arterial wall (1). Pseudoaneurysm of the superficial temporal artery is a rare (2, 3) clinical entity that typically occurs after a blunt trauma (1, 4–6) to the frontotemporal region (5). There are reports of such traumatic pseudoaneurysms of superficial temporal artery (1, 2, 6–9), but within the context of craniotomy as a cause of pseudoaneurysm only few cases (2) have been described throughout the world. We present a case of pseudoaneurysm of the superficial temporal artery emerging after craniotomy for excision of meningioma. To our knowledge, this is the first case of superficial temporal artery pseudoaneurysm developing after craniotomy (for meningioma excision) from Pakistan. We have discussed the etiology, clinical presentation, diagnosis, and treatment with reference to previously reported cases.

## Case Report

A 45-year-old male presented with a complaint of a headache lasting 3 months. On fundoscopy, he had papilledema. A CT Scan Brain revealed a large space occupying lesion arising from the sphenoid ridge along with hyperostosis. Brain MRI with contrast showed a homogenously enhancing lesion with significant mass effect. Radiological findings were consistent with meningioma.

After angioembolization, frontotemporal craniotomy was done and the lesion was excised completely. The patient had a smooth recovery in post-operative phase and he was discharged 10 days after surgery.

**Figure 1:**
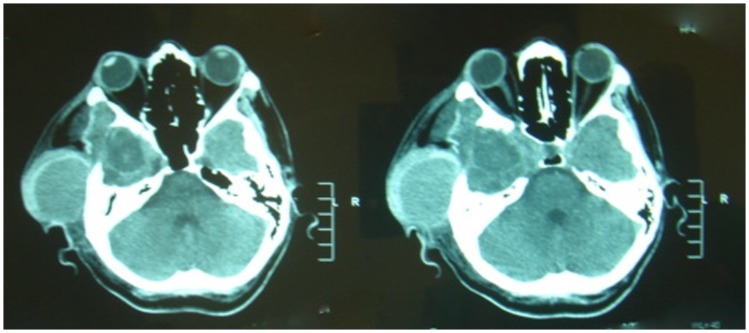
**CT scan showing superficial temporal artery pseudoaneurysm**.

**Figure 2:**
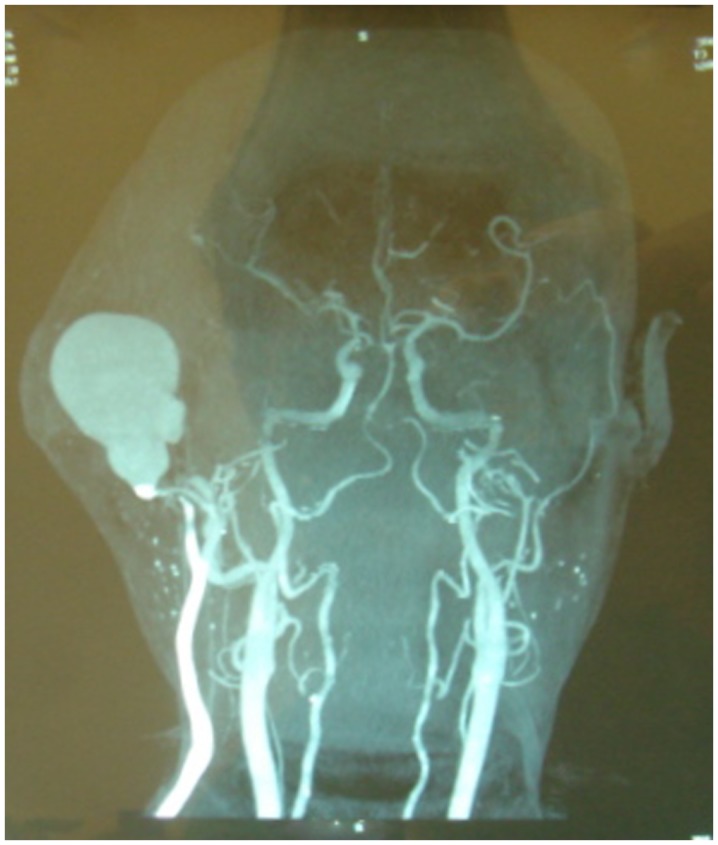
**CT angiogram**.

**Figure 3:**
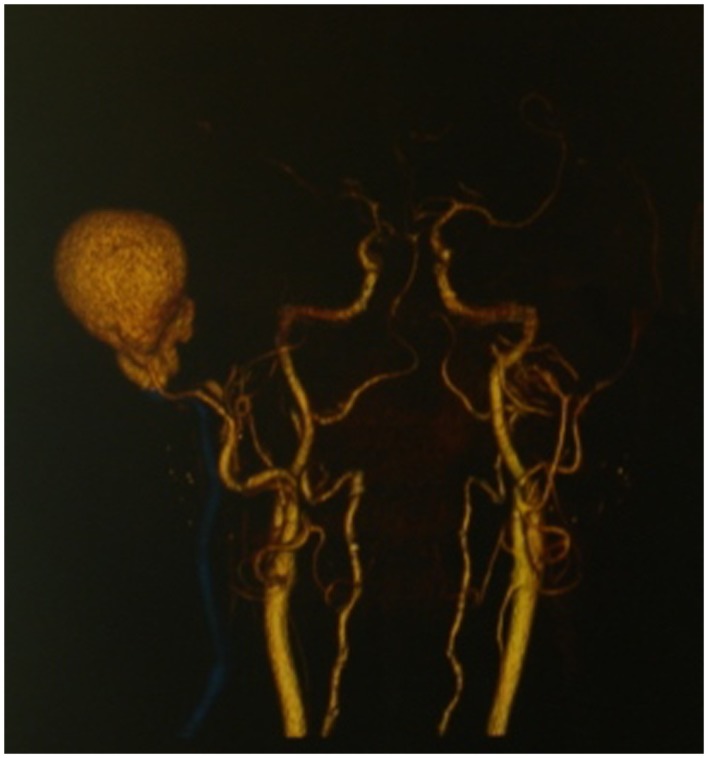
**3D reconstruction of pseudoaneurysm**.

During his follow up 1 month after surgery, he presented with a diffuse swelling of craniotomy flap, although the edges of wound were healthy. The swelling was *diffusely* homogenous, normal in color, pulsatile in nature and indolent (Figure 1). With the suspicion of pseudoaneurysm, carotid angiography was performed which revealed a large pseudoaneurysm arising from branch of the superficial temporal artery. He had a redo surgery for excision of pseudoaneurysm (Figures 2 and 3). At the base of pseudoaneurysm, there was free end of superficial temporal artery, which was coagulated and ligated. Subsequent follow up did not reveal any recurrence of the tumor or pseudoaneurysm.

## Discussion

The first case of superficial temporal artery pseudoaneurysm was described by Thomas Bartholin (1, 2, 4, 6) in 1740 and was the result of blunt trauma (2). The most common etiology of superficial temporal artery pseudoaneurysm is blunt trauma (1, 4–6, 10) accounting for 75% (4) to 95% (10) of cases; penetrating injury or iatrogenic cases were responsible for the rest of cases (10). STA pseudoaneurysms due to iatrogenic injury have been reported to occur after cyst removal, temporomandibular joint excision arthroplasty, punch hair grafting, and craniotomies (5). However, craniotomy as a cause of pseudoaneurysm of STA is extremely rare (6–8). Of the few craniotomies reported as a cause in literature, the majority were done for aneurysmal clipping (2). However, in this report we present a case of craniotomy for meningioma excision leading to STA pseudoaneurysms. Since 1985, when Rousseaux et al reported a case of an STA pseudoaneurysm that developed following a craniotomy for frontal lobe meningioma resection (9), no such cases have been reported in literature.

The anterior branch of the STA is most vulnerable to injury from blunt trauma due to its superficial course and close proximity to the underlying bony structures (1, 4, 10). However, it was affected as a result of craniotomy. Multiple aneurysms are possible but uncommon (1, 5). True aneurysms of the STA are also reported but are extremely rare (1, 2, 5). Regarding the patho physiologic mechanism of pseudoaneurysm formation, there is a consensus in the literature on occurrence of some kind of penetrating trauma during the surgery (2) by skin incision, a pin head-holder, thread removal and subcutaneous drains (4). In our case, we believe a needle injury to the STA during subcutaneous closure resulting in slow bleeding and pseudoaneurysm formation.

The time period from craniotomy to pseudoaneurysm in the literature varied between 4 days and 3 months (7), and in our case it was 1 month post-operatively. The typical history involves trauma or surgery to the temporal region (5, 7) and subsequent development of a pulsatile, indolent or expanding swelling which may be associated with headache (4, 7). Other neurologic symptoms are not always present but may include facial pain, dizziness, ear discomfort, or facial droop due to cranial nerve VII compression (1). In some cases, pulsation may be absent if there is complete thrombosis of the aneurysmal sac (10). On examination, a compressible, tender, pulsatile mass over the superior temporal line is usually apparent and occasionally bruits may be ausculatated (1, 5, 7).

Differential diagnoses for STA pseudoaneurysm include vascular tumor, arteriovenous fistula, meningeal artery aneurysm with bony erosion, subcutaneous lipoma, abscess, and localized hematoma (1–5, 7).

Many authors believe that superficial temporal artery pseudoaneurysm should be diagnosed clinically from history and physical examinations (1, 2, 4). However, diagnostic modalities involve invasive and non-invasive tests (2). For most cases, duplex ultrasound is currently the imaging modality of choice (1, 10), since it can provide detailed information about the vascular anatomy without incurring the risks of invasive methods or radiation. CT Scans with contrast, CT Angiography and Digital Substraction Angiography have been reported in the literature (5).

Indications for surgery are cosmetic, treat headache, and avoid pain and hemorrhage (2, 4). Surgical management under local anesthetic is appropriate and may include ligation and excision of the aneurysmal arterial segment or primary repair when the arterial injury is easily amenable to closure with sutures (1). Other treatment options for STA aneurysm have been reported, including endovascular obliteration, percutaneous endo-obliteration using coils, glue, or ethylene vinyl alcohol copolymer (3). Percutaneous thrombin injection as a treatment has been described with controversies (6). Recurrence rate after surgery have been documented as rare (3). In our patient surgical excision of pseudoaneurysm was carried out with no recurrence post-surgery.

## Conclusion

This case is intended to caution surgeons dealing with craniotomy of this rare complication. A post-craniotomy temporal mass should be considered a STA pseudoaneurysm until proven otherwise, and needle decompression should not be attempted. Surgical excision of the pseudoaneurysm of superficial temporal artery results in a good clinical outcome.

## Informed Consent

The patient to which the case study refers provided oral and written consent to be featured in this article.

## Conflict of Interest Statement

The authors declare that the research was conducted in the absence of any commercial or financial relationships that could be construed as a potential conflict of interest.
